# Supplementary material for: Benchmarking for Bayesian Reinforcement Learning
Source: PLoS One. 2016 Jun 15;11(6):e0157088. doi: 10.1371/journal.pone.0157088 (PMC4909278; doi:10.1371/journal.pone.0157088)
Supplement: S1 File — (PDF) [file pone.0157088.s001.pdf]

Benchmarking for Bayesian Reinforcement Learning

Michael Castronovo[1◑*], Damien Ernst[1‡], Adrien Couëtoux[1‡], Raphael Fonteneau[1◑]

1 Systems and Modeling, Montefiore Institute, University of Liege, Liege, Belgium

◑These authors contributed equally to this work.
‡These authors also contributed equally to this work.
* m.castronovo@ulg.ac.be

S1. Pseudo-code of the algorithms

Algorithm 1 ϵ-Greedy

procedure OFFLINE-LEARNING($p^0_{\mathcal{M}}(.)$)
    $\hat{M} \leftarrow$ "Build an initial model based on $p^0_{\mathcal{M}}(.)$"
end procedure

function SEARCH(x, h)
    {Draw a random value in $[0; 1]$}
    $r \leftarrow \mathcal{U}(0, 1)$

    if $r < \epsilon$ **then** {Random case}
        return "An action selected randomly"

    else {Greedy case}
        $\pi^*_{\hat{M}} \leftarrow$VALUE-ITERATION($\hat{M}$)
        return $\pi^*_{\hat{M}}(x)$
    end if
end function

procedure ONLINE-LEARNING(x, u, y, r)
    "Update model $\hat{M}$ w.r.t. transition $< x, u, y, r >$"
end procedure

Algorithm 2 Soft-max

procedure OFFLINE-LEARNING($p^0_{\mathcal{M}}(.)$)
    $\hat{M} \leftarrow$ "Build an initial model based on $p^0_{\mathcal{M}}(.)$"
end procedure

function SEARCH(x, h)
    {Draw a random value in $[0; 1]$}
    $r \leftarrow \mathcal{U}(0, 1)$

    {Select an action randomly, with a probability proportional to $Q^*_{\hat{M}}(x, u)$}
    $Q^*_{\hat{M}} \leftarrow$ "Compute the optimal Q-function of $\hat{M}$"
    for $1 \leq i \leq |U|$ **do**
        if $r < \sum_{j \leq i} \frac{\exp\left(Q^*_{\hat{M}}(x, u^{(j)}) / \tau\right)}{\sum_{u'} \exp\left(Q^*_{\hat{M}}(x, u') / \tau\right)}$ **then**
            return $u^{(i)}$
        end if
    end for
end function

procedure ONLINE-LEARNING(x, u, y, r)
    "Update model $\hat{M}$ w.r.t. transition $< x, u, y, r >$"
end procedure

Algorithm 3 OPPS-DS

procedure OFFLINE-LEARNING($p^0_{\mathcal{M}}(.)$)
    {Initialise the k arms of UCB1}
    for $1 \leq i \leq k$ **do**
        $M \sim p^0_{\mathcal{M}}(.)$
        $R^{\pi_i}_M \leftarrow$ "Simulate strategy π_i on MDP M over a single trajectory"
        $\mu(i) \leftarrow R^{\pi_i}_M$
        $\theta(i) \leftarrow 1$
    end for

    {Run UCB1 with a budget of β}
    for $k+1 \leq b \leq \beta$ **do**
        $a \leftarrow \arg\max_{a'} \mu(a') + \sqrt{\frac{2\log(b)}{\theta(a')}}$
        $M \sim p^0_{\mathcal{M}}(.)$
        $R^{\pi_a}_M \leftarrow$ "Simulate strategy π_a on MDP M over a single trajectory"
        $\mu(a) \leftarrow \frac{\theta(a)\mu(a)+R^{\pi_a}_M}{\theta(a)+1}$
        $\theta(a) \leftarrow \theta(a)+1$
    end for

    {Select the E/E strategy associated to the most drawn arm}
    $a^* \leftarrow \arg\max_{a'} \theta(a')$
    $\pi_{OPPS} \leftarrow \pi_{a^*}$
end procedure

function SEARCH(x, h)
    return $u \sim \pi_{OPPS}(x, h)$
end function

procedure ONLINE-LEARNING(x, u, y, r)
    "Update strategy π_{OPPS} w.r.t. transition $< x, u, y, r >$"
end procedure

Algorithm 4 BAMCP (1/2)

```
function SEARCH(x, h)
    {Develop a MCTS and compute Q(., .)}
    for 1 ≤ k ≤ K do
        M ~ p^h_M
        SIMULATE(⟨x, h⟩, M, 0)
    end for

    {Return the best action w.r.t. Q(., .)}
    return argmax_u Q(⟨x, h⟩, u)
end function

function SIMULATE(⟨x, h⟩, M, d)
    if N(⟨x, h⟩) = 0 then {New node reached}
        "Initialise N(⟨x, h⟩, u), Q(⟨x, h⟩, u)"
        u ~ π_0(⟨x, h⟩)
        "Sample x', r from model M"

        {Estimate the score of this node by using the rollout policy}
        R ← r + γ ROLLOUT(⟨x', hux'⟩, P, d)

        "Update N(⟨x, h⟩), N(⟨x, h⟩, u), Q(⟨x, h⟩, u)"
        return R
    end if

    {Select the next branch to explore}
    u ← argmax_u' Q(⟨x, h⟩, u) + c sqrt( log(N(⟨x, h⟩)) / N(⟨x, h⟩, u') )
    "Sample x', r from model M"

    {Follow the branch and evaluate it}
    R ← r + γ SIMULATE(⟨x', hux'⟩, M, d + 1)

    "Update N(⟨x, h⟩), N(⟨x, h⟩, u), Q(⟨x, h⟩, u)"
    return R
end function
```

Algorithm 5 BAMCP (2/2)

```
procedure ROLLOUT(⟨x, h⟩, M, d)
    if γ^d R_max < ϵ then {Truncate the trajectory if precision ϵ has been reached}
        return 0
    end if

    {Use the rollout policy to choose the action to perform}
    u ∼ π_0(x, h)

    {Simulate a single transition from M and continue the rollout process}
    y ∼ P_M
    r ← ρ_M(x, u, y)
    return r + γ ROLLOUT(⟨y, huy⟩, M, d + 1)
end procedure

procedure ONLINE-LEARNING(x, u, y, r)
    "Update the posterior w.r.t. transition < x, u, y, r >"
end procedure
```

Algorithm 6 BFS3

function SEARCH(x, h)
  {Update the current Q-function}
  $M_{mean} \leftarrow$ "Compute the mean MDP of $p^t_{\mathcal{M}}(.)$."
  for all $u \in \text{U}$ **do**
    for $1 \leq i \leq C$ **do**
      {Draw y and r from the mean MDP of the posterior}
      $y \sim P_{M_{mean}}$
      $r \leftarrow \rho_M(x, u, y)$

      {Update the Q-value in (x, u) by using $FSSS$ algorithm}
      $Q(x, u) \leftarrow Q(x, u) + \frac{1}{C}\left[r + \gamma \text{ FSSS}(y, d, t)\ \right]$
    end for
  end for

  {Return the action u with the maximal Q-value in x}
  return $\arg\max_u Q(x, u)$
end function

Algorithm 7 FSSS (1/2)

function FSSS(x, d, t)
  {Develop a MCTS and compute bounds on $V(x)$}
  for $1 \leq i \leq t$ **do**
    ROLLOUT$(s, d, 0)$
  end for

  {Make an optimistic estimation of $V(x)$}
  $\hat{V}(x) \leftarrow \max_u U_d(x, u)$
  return $\hat{V}(x)$
end function

Algorithm 8 FSSS (2/2)

procedure ROLLOUT(x, d, l)
if $d = l$ **then** {Stop when reaching the maximal depth}
return
end if
if $\neg Visited_d(x)$ **then** {New node reached}
{Initialise this node}
for all $u \in U$ **do**
"Initialise $N_d(x, u, x')$,$R_d(x, u)$"
for $1 \leq i \leq C$ **do**
"Sample x', r from M"
"Update $N_d(x, u, x')$,$R_d(x, u)$"
if $\neg Visited_d(x')$ **then**
$U_{d+1}(x'), L_{d+1}(x') = V_{max}, V_{min}$
end if
end for
end for
{Back-propagate this node's information}
BELLMAN-BACKUP(x, d)
$Visited_d(x) \leftarrow$true
end if
{Select an action and simulate a transition optimistically}
$u \leftarrow \arg\max_u U_d(x, u)$
$x' \leftarrow \arg\max_{x'} \big(U_{d+1}(x') - L_{d+1}(x')\big) N_d(x, u, x')$
{Continue the rollout process and back-propagate the result}
ROLLOUT$(x', d, l + 1)$
BELLMAN-BACKUP(x, d)
return
end procedure

Algorithm 9 SBOSS (1/2)

function SEARCH(x, h)
  {Compute the transition matrix of the mean MDP of the posterior}
  $M_{mean} \leftarrow$ "Compute the mean MDP of $p^t_{\mathcal{M}}(.)$."
  $P_t \leftarrow P_{M_{mean}}$

  {Update the policy to follow if necessary}
  $\forall(x,u) : \Delta(x,u) = \sum_{y \in X} \frac{|P_t(x,u,y) - P_{lastUpdate}(x,u,y)|}{\sigma(x,u,y)}$
  if $t = 1$ or $\exists(x', u') : \Delta(x', u') > \delta$ **then**
    {Sample some transition vectors for each state-action pair}
    $S \leftarrow \{\}$
    for all $(x, u) \in X \times U$ **do**
      {Compute the number of transition vectors to sample for (x, u)}
      $K_t(x,u) \leftarrow \max_y \left\lceil \frac{\sigma^2(x,u,y)}{\epsilon} \right\rceil$

      {Sample $K_t(x,u)$ transition vectors from $< x, u >$, sampled from the posterior}
      for $1 \leq k \leq K_t(x,u)$ **do**
        $S \leftarrow S \cup$ "A transition vector from $< x, u >$, sampled from the posterior"
      end for
    end for

    $M^{\#} \leftarrow$ "Build a new MDP by merging all transitions from S"
    $\pi^*_{M^{\#}} \leftarrow$VALUE-ITERATION$(M^{\#})$
    $\pi_{SBOSS} \leftarrow$FIT-ACTION-SPACE$(\pi^*_{M^{\#}})$
    $P_{lastUpdate} \leftarrow P_t$
  end if

  {Return the optimal action in x w.r.t. π_{SBOSS}}
  return $u \sim \pi_{SBOSS}(x)$
end function

Algorithm 10 SBOSS (2/2)

function FIT-ACTION-SPACE$(\pi^*_{M^{\#}})$
  for all $x \in X$ **do**
    $\pi(x) \leftarrow \pi^*_{M^{\#}}(x) \mod |U|$
  end for

  return π
end function

procedure ONLINE-LEARNING(x, u, y, r)
  "Update the posterior w.r.t. transition $< x, u, y, r >$"
end procedure

Algorithm 11 BEB

procedure SEARCH(x, h)
    $M \leftarrow$ "Compute the mean MDP of $p^t_{\mathcal{M}}(.)$."

    {Add a bonus reward to all transitions}
    for $< x, u, y > \in \mathcal{X} \times \mathcal{U} \times \mathcal{X}$ **do** $\rho_M(x, u, y) \leftarrow \rho_M(x, u, y) + \frac{\beta}{c^{(t)}_{<x,u,y>}}$
    end for

    {Compute the optimal policy of the modified MDP}
    $\pi^*_M \leftarrow$VALUE-ITERATION(M)

    {Return the optimal action in x w.r.t. π^*_M}
    return $u \sim \pi^*_M(x)$
end procedure

procedure ONLINE-LEARNING(x, u, y, r)
    "Update the posterior w.r.t. transition $< x, u, y, r >$"
end procedure
