# Supplementary material for: Benchmarking for Bayesian Reinforcement Learning
Source: PLoS One. 2016 Jun 15;11(6):e0157088. doi: 10.1371/journal.pone.0157088 (PMC4909278; doi:10.1371/journal.pone.0157088)
Supplement: S2 File — (PDF) [file pone.0157088.s002.pdf]

Benchmarking for Bayesian Reinforcement Learning

Michael Castronovo[1◑*], Damien Ernst[1‡], Adrien Couëtoux[1‡], Raphael Fonteneau[1◑]

1 Systems and Modeling, Montefiore Institute, University of Liege, Liege, Belgium

◑These authors contributed equally to this work.
‡These authors also contributed equally to this work.
* m.castronovo@ulg.ac.be

S2. MDP distributions in detail

In this section, we describe the MDPs drawn from the considered distributions in more detail. In addition, we also provide a formal description of the corresponding $\boldsymbol{\theta}$ (parameterising the FDM used to draw the transition matrix) and ρ_M (the reward function).

1 Generalised Chain distribution

On those MDPs, we can identify two possibly optimal behaviours:

- The agent tries to move along the chain, reaches the last state, and collect as many rewards as possible before returning to State 1;
- The agent gives up to reach State 5 and tries to return to State 1 as often as possible.

1.1 Formal description

$$X = \{1, 2, 3, 4, 5\},\ U = \{1, 2, 3\}$$

$$\forall u \in U :$$

$$\begin{aligned}
\theta^{GC}_{1,u} &= [1, 1, 0, 0, 0] \\
\theta^{GC}_{2,u} &= [1, 0, 1, 0, 0] \\
\theta^{GC}_{3,u} &= [1, 0, 0, 1, 0] \\
\theta^{GC}_{4,u} &= [1, 0, 0, 0, 1] \\
\theta^{GC}_{5,u} &= [1, 1, 0, 0, 1]
\end{aligned}$$

$$\forall x, u \in X \times U :$$

$$\begin{aligned}
\rho^{GC}(x, u, 1) &= 2.0 \\
\rho^{GC}(x, u, 5) &= 10.0 \\
\rho^{GC}(x, u, y) &= 0.0,\ \forall y \in X \setminus \{1, 5\}
\end{aligned}$$

2 Generalised Double-Loop distribution

Similarly to the GC distribution, we can also identify two possibly optimal behaviours:

- The agent enters the “good” loop and tries to stay in it until the end;
- The agent gives up and chooses to enter the “bad” loop as frequently as possible.

2.1 Formal description

$$X = \{1,2,3,4,5,6,7,8,9\},\ U = \{1,2\}$$

$$\forall u \in U:$$

$$\theta^{GDL}_{1,u} = [0,1,0,0,0,1,0,0,0]$$
$$\theta^{GDL}_{2,u} = [0,0,1,0,0,0,0,0,0]$$
$$\theta^{GDL}_{3,u} = [0,0,0,1,0,0,0,0,0]$$
$$\theta^{GDL}_{4,u} = [0,0,0,0,1,0,0,0,0]$$
$$\theta^{GDL}_{5,u} = [1,0,0,0,0,0,0,0,0]$$
$$\theta^{GDL}_{6,u} = [1,0,0,0,0,0,1,0,0]$$
$$\theta^{GDL}_{7,u} = [1,0,0,0,0,0,0,1,0]$$
$$\theta^{GDL}_{8,u} = [1,0,0,0,0,0,0,0,1]$$
$$\theta^{GDL}_{9,u} = [1,0,0,0,0,0,0,0,0]$$

$$\forall u \in U:$$

$$\rho^{GDL}(5,u,1) = 1.0$$
$$\rho^{GDL}(9,u,1) = 2.0$$
$$\rho^{GDL}(x,u,y) = 0.0,\ \forall x \in X,\ \forall y \in X : y \neq 1$$

3 Grid distribution

MDPs drawn from the Grid distribution are 2-dimensional grids. Since the agents considered do not manage multi-dimensional state spaces, the following bijection was defined:

$$\{1,2,3,4,5\} \times \{1,2,3,4,5\} \rightarrow X = \{1,2,\cdots,25\} : n(i,j) = 5(i-1)+j$$

where i and j are the row and column indexes of the cell on which the agent is.

When the agent reaches the **G** cell (in $(5,5)$), it is directly moved to $(1,1)$, and will perceive its reward of 10. In consequence, State $(5,5)$ is not reachable.

To move inside the Grid, the agent can perform four actions: $U = \{up, down, left, right\}$. Those actions only move the agent to one adjacent cell. However, each action has a certain probability to fail (depending on the cell on which the agent is). In case of failure, the agent does not move at all. Besides, if the agent tries to move out of the grid, it will not move either. Discovering a reliable (and short) path to reach the **G** cell will determine the success of the agent.

3.1 Formal description

$$X = \{1,2,\cdots,25\}, U = \{up,\ down,\ left,\ right\}$$

$$\forall (i,j) \in \{1,2,3,4,5\} \times \{1,2,3,4,5\}$$
$$\forall (k,l) \in \{1,2,3,4,5\} \times \{1,2,3,4,5\} :$$

$$
\begin{aligned}
\theta^{Grid}_{n(i,j),u} \quad & (n(i,j)) &&= 1,\ \forall u \in U \\
\\
\theta^{Grid}_{n(i,j),up} \quad & (n(i-1,j)) &&= 1, (i-1) \geq 1 \\
\theta^{Grid}_{n(i,j),down} \quad & (n(i+1,j)) &&= 1, (i+1) \leq 5, (i,j) \neq (4,5) \\
\theta^{Grid}_{n(i,j),left} \quad & (n(i,j-1)) &&= 1, (j-1) \geq 1 \\
\theta^{Grid}_{n(i,j),right} \quad & (n(i,j+1)) &&= 1, (j+1) \leq 5, (i,j) \neq (5,4) \\
\\
\theta^{Grid}_{n(4,5),down} \quad & (n(1,1)) &&= 1 \\
\theta^{Grid}_{n(5,4),right} \quad & (n(1,1)) &&= 1 \\
\\
\theta^{Grid}_{n(i,j),u} \quad & (n(k,l)) &&= 0, else
\end{aligned}
$$

$$
\begin{aligned}
\rho^{Grid}((4,5), down, (1,1)) &= 10.0 \\
\rho^{Grid}((5,4), right, (1,1)) &= 10.0 \\
\\
\rho^{Grid}((i,j), u, \quad (k,l)) &= 0.0,\ \forall u \in U
\end{aligned}
$$
