# Supplementary material for: Benchmarking for Bayesian Reinforcement Learning
Source: PLoS One. 2016 Jun 15;11(6):e0157088. doi: 10.1371/journal.pone.0157088 (PMC4909278; doi:10.1371/journal.pone.0157088)
Supplement: S3 File — (PDF) [file pone.0157088.s003.pdf]

Benchmarking for Bayesian Reinforcement Learning

Michael Castronovo[1◑*], Damien Ernst[1‡], Adrien Couëtoux[1‡], Raphael Fonteneau[1◑]

1 Systems and Modeling, Montefiore Institute, University of Liege, Liege, Belgium

◑These authors contributed equally to this work.
‡These authors also contributed equally to this work.
* m.castronovo@ulg.ac.be

S3. Paired sampled Z-test

Let π_A and π_B be the two agents we want to compare. We played the two agents on the same N MDPs, denoted by $M_1, \cdots, M_N$. Let $R^{\pi_A}_{M_i}$ and $R^{\pi_B}_{M_i}$ be the scores we observed for the two agents on M_i.

Step 1 - Hypothesis

We compute the mean and the standard deviation of the differences between the two sample sets, denoted by $\bar{x}_d$ and $\bar{s}_d$, respectively.

$$\bar{x}_d = \frac{1}{N}\sum_{i=1}^{N} R^{\pi_A}_{M_i} - R^{\pi_B}_{M_i}$$

$$\bar{s}_d = \frac{1}{N}\sum_{i=1}^{N} (\bar{x}_d - (R^{\pi_A}_{M_i} - R^{\pi_B}_{M_i}))^2$$

If $N \geq 30$, $\bar{s}_d$ is a good estimation of σ_d, the standard deviation of the differences between the two populations ($\bar{s}_d \approx \sigma_d$). In order words, σ_d is the standard deviation we should observe when testing the two algorithms on a number of MDPs tending towards infinity. This was always the case in our experiments.

We now set Hypothesis H_0 and Hypothesis H_α:

$$H_0 : \mu_d = 0$$

$$H_\alpha : \mu_d > 0$$

Our goal is to determine if μ_d, the mean of the differences between the two populations, is equal or greater than 0. More expressly, we want to know if the differences between the two agents' performances is significant (H_α is correct) or not (H_0 correct). Only one of those hypotheses can be true.

Step 2 - Test statistic

The test statistic consists to compute a certain value Z:

$$Z = \frac{\bar{x_d}}{\sigma_d \Big/ \sqrt{N}}$$

This value will help us to determine if we should accept (or reject) hypothesis H_α.

Step 3 - Rejection region

Assuming we want our decision to be correct with a probability of failure of α, we will have to compare Z with Z_α, a value of a Gaussian curve. If $Z > Z_\alpha$, it means we are in the rejection region (R.R.) with a probability equal to $1 - \alpha$. For a confidence of 95%, Z_α should be equal to 1.645 as show by Fig A.

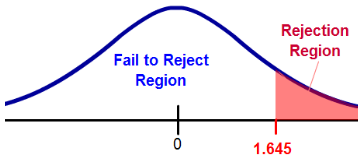

Fig A. Rejection region (with a confidence of 95%)

Being in the R.R. means we have to reject Hypothesis H_0 (and accept Hypothesis H_α). In the order case, we have to accept Hypothesis H_0 (and reject Hypothesis H_α).

Step 4 - Decision

At this point, we have either accepted Hypothesis H_0 or Hypothesis H_α.

- **Accepting Hypothesis H_0 ($Z < Z_\alpha$)**: The two algorithms π_A and π_B are not significantly different.
- **Accepting Hypothesis H_α ($Z \geq Z_\alpha$)**: The two algorithms π_A and π_B are significantly different. Therefore, the algorithm with the greatest mean is definitely better with 95% confidence.
